# A systematic review investigating the safety and efficacy of intravenous iron in people with ND-CKD who are iron deficient but not anaemic

**DOI:** 10.1093/ckj/sfag172

**Published:** 2026-06-08

**Authors:** Sebastian Spencer, Megan Skidmore, Rosa Maeve McGing, Sunil Bhandari

**Affiliations:** Hull University Teaching Hospitals NHS Trust, Hull, UK; Hull York Medical School, Hull, UK; Northern Care Alliance (NCA), NHS Foundation Trust, Manchester, UK; Haxby Group Medical Practice, Hull, UK; Hull University Teaching Hospitals NHS Trust, Hull, UK; Hull York Medical School, Hull, UK

**Keywords:** anaemia, chronic kidney disease, iron deficiency, quality of life

## Abstract

**Background:**

The role of intravenous (IV) iron in non-dialysis-dependent chronic kidney disease (ND-CKD) without anaemia remains uncertain. Iron deficiency (ID) in CKD is common and associated with impaired mitochondrial function, fatigue, cognitive decline, and restless legs syndrome, even when haemoglobin (Hb) levels are normalized. ID is linked to cardiovascular (CV) outcomes and increased hospitalization risk. Guidelines are inconsistent, and the evidence base is limited.

**Methods:**

Databases were searched for randomized controlled trials (RCTs) and cohort studies involving adults with ND-CKD (estimated glomerular filtration rate [eGFR] < 60) receiving IV iron compared with oral iron, placebo, or no treatment. Primary outcome was change in iron indices. Secondary outcomes: CV events, infections, physical function, symptoms, cognition, and quality of life (QoL).

**Results:**

Six studies met the inclusion criteria. In pooled analyses restricted to RCTs, IV iron significantly improved TSAT [mean difference (MD) +7.48%, 95% CI 5.64–9.32; *P* < .001; *I*² = 0.0%] and ferritin (MD +208.83 ng/ml, 95% CI 128.39–289.26; *P* = .004; *I*² = 75.9%). IV iron was associated with a numerically greater increase in Hb, although this did not reach statistical significance (MD +5.33 g/l, 95% CI −1.18 to 11.84; *P* = .080; *I*² = 57.4%). No significant effect was observed on 6-minute walk test distance (MD +23.99 m, 95% CI −77.44 to 125.41; *P* = .506; *I*² = 83.0%). QoL outcomes showed no significant difference in the primary standardized mean difference (SMD) meta-analysis (SMD −0.23, 95% CI −1.44 to 0.98; *P* = .587; *I*² = 83.2%). Adverse events, CV outcomes and infections were sparsely reported, with no consistent safety signal identified.

**Conclusions:**

IV iron improves biochemical markers of ID in ND-CKD without anaemia, with no clear signal of harm. However, evidence does not demonstrate measurable improvements in physical function or QoL.

KEY LEARNING POINTS **What was known:**

- Intravenous iron is established in haemodialysis, but its role in iron-deficient, non-anaemic non-dialysis CKD remained uncertain, particularly regarding safety, dose strategies, and whether biochemical improvement translates into patient benefit.
- Iron deficiency in CKD can cause fatigue, reduced exercise tolerance, cognitive effects and restless legs syndrome even without anaemia, yet treatment is often deferred until anaemia develops.
- Existing guidance was inconsistent and evidence was limited by small studies, heterogeneous definitions, varied dosing regimens, and inconsistent reporting of patient-reported and functional outcomes, creating uncertainty for practice.

**This study adds:**

- This review shows that intravenous iron in non-anaemic non-dialysis CKD is biologically effective, consistently improving ferritin, transferrin saturation and Hb across studies.
- Despite biochemical correction, there was no convincing evidence of benefit in physical function, symptoms, or QoL, and pooled analysis of 6-minute walk distance showed no significant improvement.
- No clear safety signal emerged, but short follow-up, low event numbers, and small study sizes mean long-term safety and cumulative dosing effects remain incompletely characterized.

**Potential impact:**

- Current evidence does not support routine use of intravenous iron in non-anaemic non-dialysis CKD solely to improve symptoms, function, or QoL, despite reliable correction of iron indices.
- These findings support conservative guideline positions and suggest treatment decisions should remain selective, particularly where ID is profound or specific symptoms are being targeted.
- Future trials should use standardized, kidney-relevant patient-centred outcomes, longer follow-up, and clearer phenotyping of ID without anaemia to identify which subgroups may genuinely benefit.

## INTRODUCTION

Intravenous (IV) iron is established as standard care for patients receiving maintenance haemodialysis (HD). By contrast, its role in people with non-dialysis-dependent chronic kidney disease (ND-CKD) who have iron deficiency (ID) but are not anaemic remains uncertain with respect to safety, optimal dosing, and patient-centred outcomes. ID is most commonly associated with anaemia, a common complication of CKD, with prevalence increasing from ∼8% to 10% in stage 1 CKD to >50% by stage 5/end-stage kidney disease [1]. In England, CKD affects ∼4.2% of the population [2]. ID is a recognized and symptomatic complication, typically presenting with lethargy, dyspnoea, headache, and restless legs syndrome (RLS) [3, 4].

ID in CKD is clinically important even without anaemia. Beyond erythropoiesis, iron is essential for mitochondrial function, energy metabolism, immune regulation, and neurocognitive processes [5, 6]. Deficiency is associated with fatigue, reduced exercise tolerance, cognitive impairment, and RLS, adversely affecting quality of life (QoL) [7–9]. ID is also linked to adverse cardiovascular (CV) outcomes and increased hospitalisation risk, independent of haemoglobin (Hb) [10–12]. Despite this, ID without anaemia is often overlooked, with treatment deferred until anaemia develops, highlighting the need to consider ID as a therapeutic target.

Globally, over 1.2 million people die from CKD annually, and patients are up to 10 times more likely to die prematurely than to progress to kidney failure requiring kidney replacement therapy [13]. Symptom burden and impaired QoL are compounded by comorbidities and treatment side effects, yet patient-centred outcomes remain inconsistently reported. The Standardised Outcomes in Nephrology—Chronic Kidney Disease (SONG-CKD) initiative aims to standardize outcome reporting across ND-CKD trials [14].

NICE guidance (2015 update) [15] recommends hypochromic red cells (HRC > 6%) as a first-line diagnostic for ID in CKD due to high sensitivity (82%) and specificity (95%) when processed within 6 h [16, 17]. Where unavailable, reticulocyte haemoglobin content (CHr < 29 pg) or reticulocyte haemoglobin equivalent (RET-He) may be used; however, serum ferritin (SF <100 μg/l) and/or transferrin saturation (TSAT < 20%) are most commonly used. Interpretation requires caution: ferritin is an acute-phase reactant and may be elevated in inflammation [18], while TSAT varies with serum iron, diet and circadian rhythm [19–22].

In HD, the PIVOTAL trial showed that proactive high-dose iron sucrose (400 mg monthly unless SF > 700 μg/l or TSAT ≥ 40%) reduced death or major CV events compared with a reactive low-dose regimen, while lowering ESA use and transfusion rates without increasing infections or hospitalizations [23]. Extrapolation to ND-CKD without anaemia is uncertain, and guidance is inconsistent. NICE [15] provides no specific recommendations, KDIGO [24] suggests treatment only in profound deficiency, and UKKA recommends IV iron primarily in those with concomitant heart failure (HF), with consideration in others for symptom relief [25–28].

Evidence from HF trials (IRONMAN, CONFIRM-HF, AFFIRM-AHF, FAIR-HF, HEART-FID) supports IV iron in symptomatic HF with ID [29–33]. As ND-CKD and HF frequently co-exist, these may include informative ND-CKD subgroups (eGFR < 60 ml/min/1.73 m²). However, ND-CKD studies (FIND-CKD, NIMO-CKD, Iron & Heart, Iron & Muscle) [34–39] leave uncertainty regarding benefits, risks, and patient-centred outcomes when Hb is non-anaemic.

*Objectives*. This systematic review and meta-analysis evaluated the efficacy and safety of IV iron for ID in adults with ND-CKD without anaemia. The primary objective was to assess effects on iron indices (ferritin, TSAT, CHr, %HRC, haematocrit). Secondary objectives included CV outcomes, infections, physical function, symptom burden (including RLS), cognition, and QoL. ND-CKD subgroup data (eGFR < 60 ml/min/1.73 m²) from HF trials were also examined.

*Definitions*. ND-CKD was defined as CKD with an eGFR < 60 ml/min/1.73 m² in adults not receiving dialysis and without a functioning transplant. ‘Not anaemic’ was defined as a Hb 110–150 g/l; ID as ≥1 of: ferritin < 100 μg/l; or ferritin >100 μg/l and TSAT < 20%; or CHr < 31 pg; or HRC > 6%, aligned UKKA guidance (2024) [25].

## MATERIALS AND METHODS

This review was conducted in accordance with the *Cochrane Handbook for Systematic Reviews of Interventions* and is reported in line with PRISMA 2020 [40]. The protocol is registered on PROSPERO (CRD42025636293) [41].

### Eligibility criteria

We included randomized controlled trials (RCTs) and observational cohort studies (prospective or retrospective) involving adults (≥18 years) with ND-CKD (eGFR < 60 ml/min/1.73 m²) not receiving kidney replacement therapy. For HF trials, only ND-CKD subgroups (eGFR < 60 ml/min/1.73 m²) were eligible. Interventions comprised any IV iron formulation (e.g. iron sucrose, ferric carboxymaltose, ferric derisomaltose/iron isomaltoside). Comparators were oral iron, placebo, or no iron. Case reports, reviews, editorials, and abstracts without extractable data were excluded.

- *Primary outcome:* change in iron indices (ferritin, TSAT, CHr, %HRC; haematocrit where reported) from baseline to last follow-up.
- *Secondary outcomes:* CV events, infections, physical function, symptom burden (including RLS), cognition, and health-related QoL.

### Information sources and search strategy

MEDLINE, Embase, Cochrane Central Register of Controlled Clinical Trials (CENTRAL), HMIC, and AMED were searched from inception to 5 May 2026, restricted to English-language publications. Grey literature was searched where available, and attempts were made to contact corresponding authors for clarification or additional data when required. The strategy combined terms for ND-CKD, iron, and IV administration, with HF terms to capture ND-CKD subgroups in HF trials. Duplicates were removed before screening.

### Study selection

**Figure 1:**
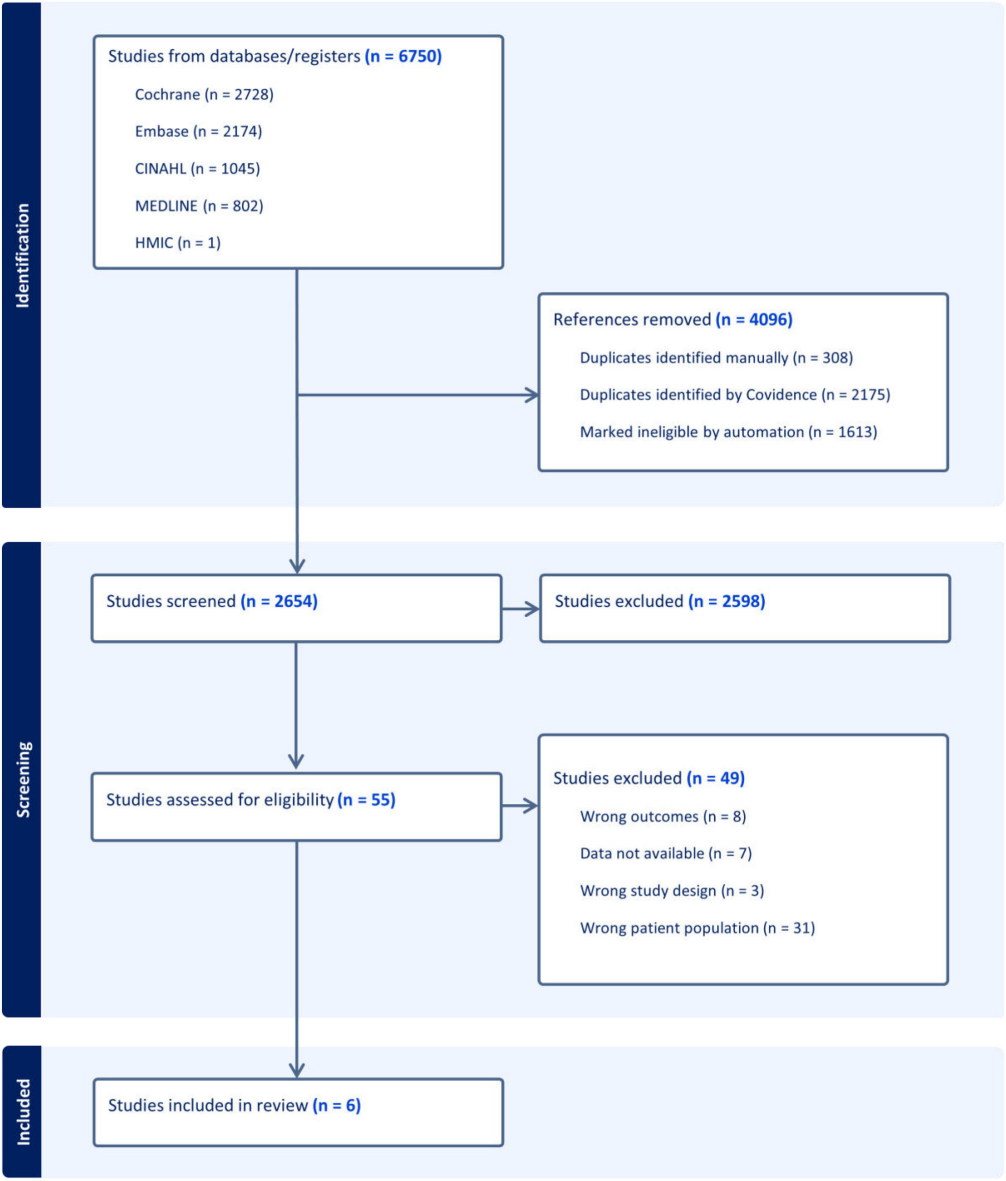
PRISMA 2020 flow diagram for study selection.

Two reviewers (S.S., M.S.) independently screened titles/abstracts and full texts, with disagreements resolved by discussion or third-reviewer adjudication (S.B.). The process is summarised in a PRISMA flow diagram (Fig. 1).

### Data extraction

Data were managed in Covidence™ (Veritas Health Innovation, Melbourne, Australia). Two reviewers independently extracted study-level information on design, setting, sample size, participant characteristics, intervention and comparator details, outcomes, adverse events, and author-reported limitations. Only ND-CKD subgroup data were extracted from HF trials. Discrepancies were resolved by consensus or SB. Missing data were recorded as ‘ND.’

### Risk of bias

Risk of bias was assessed independently by two reviewers (SS and MS) using Joanna Briggs Institute tools: the Critical Appraisal Checklist for RCTs and the Checklist for Cohort Studies [42]. Disagreements were resolved by consensus or arbitration.

### Data synthesis and analysis

Where studies were sufficiently comparable, meta-analysis was performed using R version 4.4.2 (R Project, 2024) with the *meta* and *metafor* packages. Continuous outcomes, including Hb, ferritin, TSAT, and 6-minute walk test distance, were analysed using mean difference (MD) with 95% confidence intervals (CI). QoL outcomes were analysed using standardized mean difference (SMD; Hedges’ g) because different validated instruments were used across studies. Statistical significance was set at *P* < .05.

Heterogeneity was assessed using Cochran’s Q and *I*² statistics. Random-effects models were used where substantial heterogeneity was present (*I*² > 50% or heterogeneity *P* < .05); otherwise, common-effect models were applied. In random-effects models, study weights were derived using inverse-variance weighting incorporating both within-study variance and between-study heterogeneity; therefore, larger studies did not necessarily dominate pooled estimates when heterogeneity was substantial.

For QoL analyses, one overall or most clinically relevant measure was selected from each study to avoid unit-of-analysis error and overrepresentation of studies reporting multiple subdomains. Where standard deviations were not reported, they were estimated from reported ranges or standard errors using Cochrane-recommended methods.

Quantitative meta-analysis was restricted to randomized controlled trials to minimize bias from pooling different study designs. Retrospective observational evidence was summarized narratively and was not included in pooled estimates.

### Ethics

As this review used published data, ethical approval was not required.

## RESULTS

A total of 6750 studies were found via database searches. Two thousand four hundred eighty-three duplicates were removed and 1613 were excluded as ineligible. On title and abstract screening, 2598 studies were removed and 49 were excluded after full text review. Six studies were included in this systematic review and meta-analysis (Fig. 1). A summary of included studies is listed below (Table 1). Baseline characteristics of the participants in these studies is noted in Table 2 .

**Table 1:** Summary of included studies.

Author, year, location	Research design	Participants	Intervention, comparators	Primary outcomes
Von Haehling *et al*. (2024) Germany	Randomized control trial	40 (randomized) 39 (completed study)	Ferric carboxymaltose 1000–2000 mg, Saline	ED-5D-3 L KCCQ Rates of infection Adverse events Haemoglobin TSAT Ferritin 6MWT
Kalra *et al*. (2022) UK	Randomized control trial	1137 (assigned) 730 (completed study)	Ferric derisomaltose [20 mg/kg (<50 kg), 1000 mg/20 mg/kg (50–69 kg), 20 mg/kg/2000 mg (70 kg+)], Usual care (at investigators discretion)	EQ-5D VAS EQ-5D index Cardiovascular outcomes Rates of infection Adverse events Rates of hospitalization 6MWT
Bhandari *et al*. (2021) UK	Randomized control trial	54 (no withdrawal)	Ferric derisomaltose (1000 mg), Saline (100 ml)	KD-QOL SF-36 Rates of infection Adverse events Haemoglobin TSAT Ferritin 6MWT
Greenwood *et al*. (2023) UK	Randomized control trial	75 42 (analysed)	Ferric carboxymaltose (1000 mg), Saline (100 ml)	Rates of infection Adverse events Haemoglobin TSAT Ferritin 6MWT Sit to stand test KD-QOL
McMahon *et al*. (2010) Australia	Randomized control trial	100 85 (achieved protocol) 77 (completed 12 month follow up)	Iron sucrose (100–200 mg), Ferrous Sulphate (325 mg)	Haemoglobin TSAT Ferritin
AlSahow *et al*. (2022) Kuwait	Retrospective cohort study	289	IV Iron (non-specific), Control not specified	Rates of infection Haemoglobin TSAT Rates of hospitalization

**Table 2:** Baseline characteristics of study populations.

Study	Age (years)			Gender	Serum ferritin (ng/ml)		Transferrin saturation (%)		HAEMOGLOBIN (g/L)		eGFR (ml/min/1.73 m^2^)		6 MINUTE WALK TEST (metres [m])	
	Intervention	Control	Overall		Intervention	Control	Intervention	Control	Intervention	Control	Intervention	Control	Intervention	Control
Von Haehling *et al*. (2024)	76 (8.9)	79 (7.03)		M 44%—F 56% (FCM M 33%—F 67% (Placebo)	44 (23–72)	50 (25–94)	19.9 (13.8–24.8)	16.0 (14.0–21.0)	129 (117–132)	12.0 (114–128)	40 (32–66)	59 (36–76)	308 (198–378)	325 (250–342)
Kalra *et al*. (2022)	73.2	73.5		M 75%—F 25% (FDI) M 72%—F 28% (Usual)	49 (30.0–86.0)	50 (30.0–85.0)	15 (11–20)	15 (10–19)	121 (112 -128)	121 (112–129)	51.7 (38.1–68.1)	50.1 (37.8–68.6)		
Bhandari *et al*. (2021)	61.6 (10.1)	57.8 (12.9)	59.6 (11.7)	M 49%—F 51%	64.2 (29.1)	68.4 (55.3)	22.3 (8.8)	19.7 (5.6)	131 (7.4)	126.6 (11.8)	33.2 (9.3)	29.1 (9.6)	386.6 (135.8)	414.7 (104.5)
Greenwood *et al*. (2023)	54 (16)	61 (12)	57 (14)	M 43%—F 57%	57 (54)	62 (33)	23 (12)	21 (6)	122.4 (92)	127.1 (12)	34 (12)	35 (11)	384 (195)	469 (142)
McMahon *et al*. (2010)	70	68	69.5 (58.5–74.5)	M 73%—F 27%	122 (71–176)	90 (58–150)	22 (18–26)	21 (15–24)	119 (7)	116 (12)	25 (8)	26 (11)		
AlSahow *et al*. (2022)			55.3 (12.0)	M 36.7%—F 63.3%			15.3 (5.1)		111.5 (15.0)		39.2 (6.8)			

Values are mean and SD or range.

FCM, ferric carboxymaltose; FDI, ferric derisomaltose; M, male; F, female.

### Quality assessment

**Figure 2:**
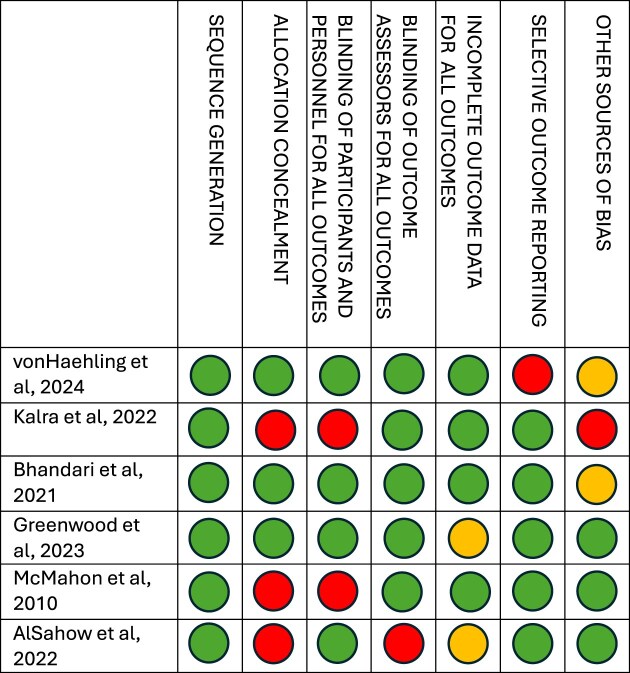
The risk of bias calculated using the revised Cochrane Risk of Bias Tool.

The risk of bias was evaluated independently by two authors (SS and MS) using the revised Cochrane Risk of Bias Tool, which was suitable for individually randomized, parallel-group trials [43] (Fig. 2). Red representing where there was deemed high risk, green low risk and orange where risk was unclear.

### Haemoglobin

**Figure 3:**
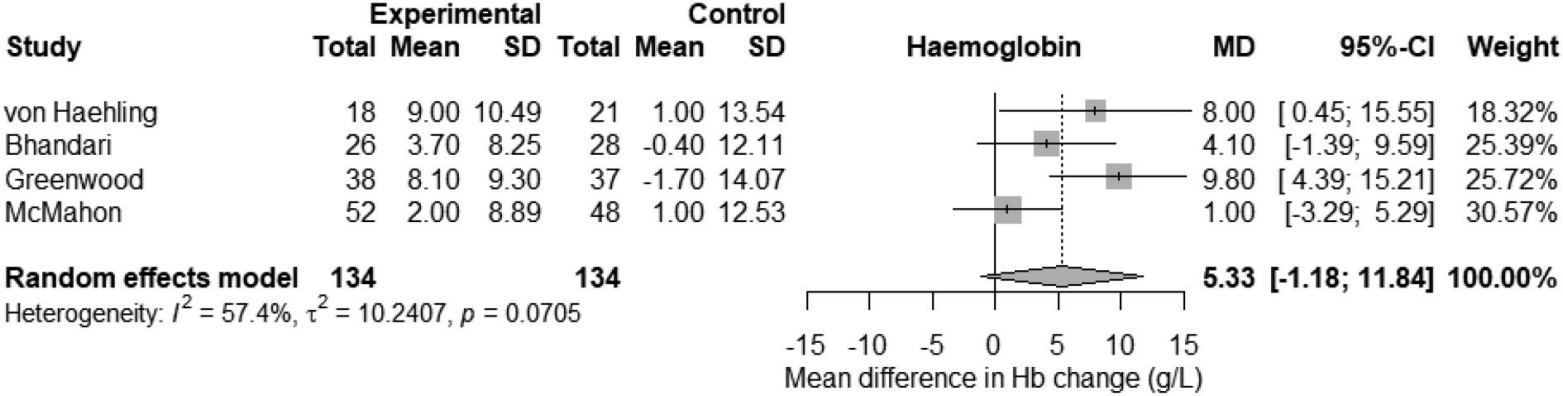
Haemoglobin mean difference (g/l).

Four studies reporting Hb outcomes were included in random-effects MD meta-analysis using change-from-baseline values. IV iron was associated with a numerically greater increase in Hb compared with control, although this did not reach statistical significance (MD 5.33 g/l, 95% CI −1.18 to 11.84; *P* = .080). Moderate heterogeneity was observed between studies (*I*² = 57.4%) (Fig. 3).

In the retrospective single-arm study by AlSahow *et al*., mean Hb increased from 111.5 g/l at baseline to 122.2 g/l following IV iron, corresponding to a mean within-group increase of 10.7 g/l. This study was not included in the comparative meta-analysis because no control group was reported.

### Serum ferritin

**Figure 4:**
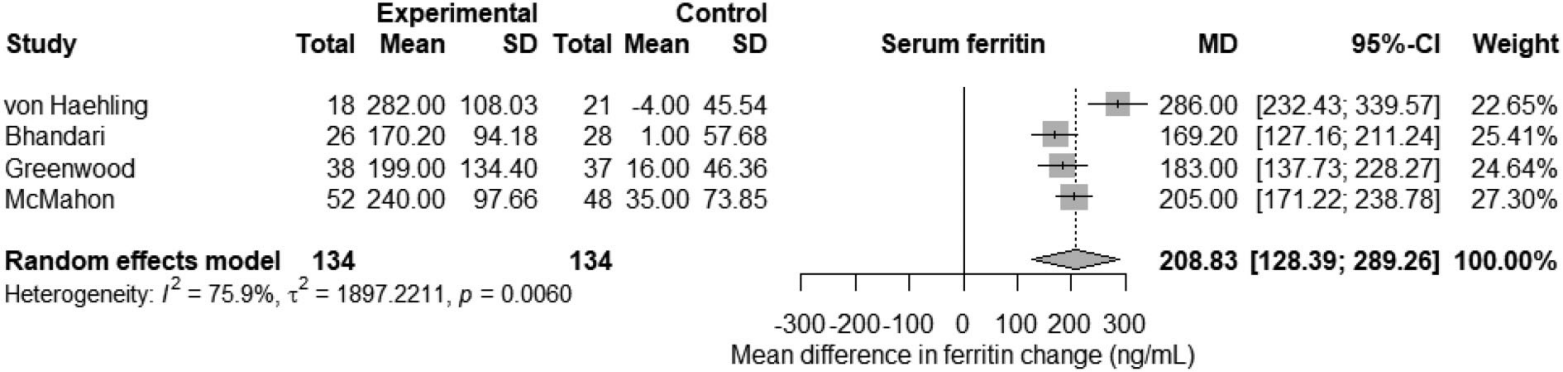
Ferritin mean difference (ng/ml).

Four studies reporting serum ferritin outcomes were included in random-effects MD meta-analysis using change-from-baseline values. IV iron significantly increased serum ferritin compared with control (MD 208.83 ng/ml, 95% CI 128.39–289.26; *P* = .004), with substantial heterogeneity observed between studies (*I*² = 75.9%) (Fig. 4).

### Transferrin saturation

**Figure 5:**
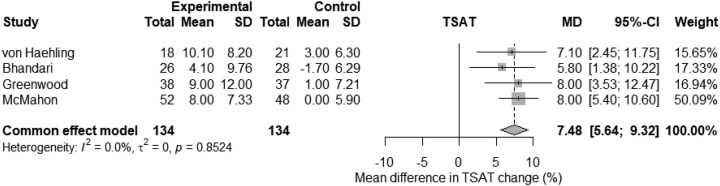
TSAT mean difference (%).

Four studies reporting TSAT outcomes were included in common-effect MD meta-analysis using change-from-baseline values. IV iron significantly increased TSAT compared with control (MD 7.48%, 95% CI 5.64–9.32; *P* < .001), with no observed statistical heterogeneity between studies (*I*² = 0%) (Fig. 5).

### 6-minute walk test

**Figure 6:**
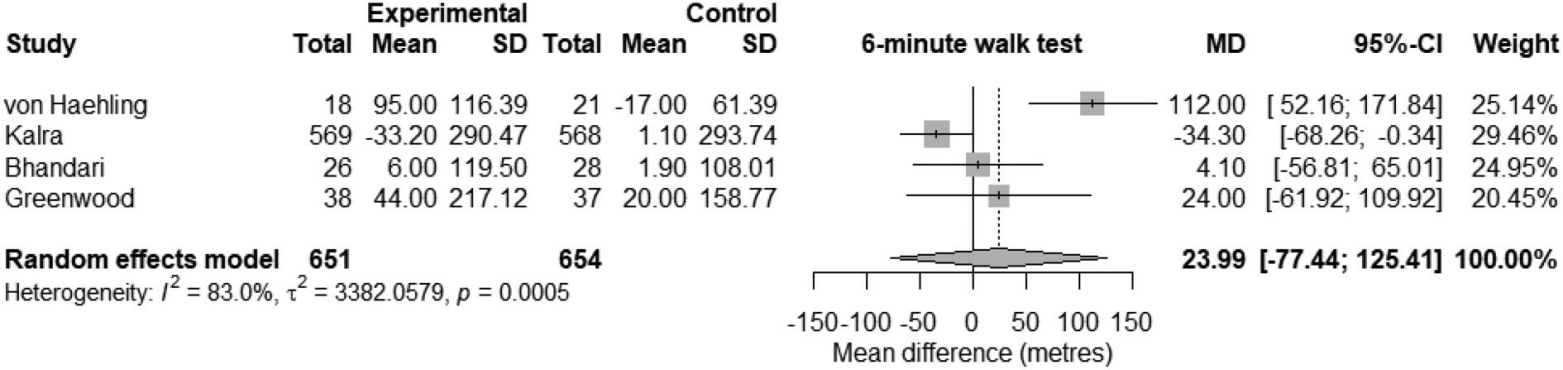
6MWT mean difference [metres (m)].

Four studies reported 6MWT outcomes and were included in random-effects MD meta-analysis using change-from-baseline values. There was no significant difference in 6MWT performance between IV iron and control groups (MD 23.99 m, 95% CI −77.44 to 125.41; *P* = .506), with substantial heterogeneity observed between studies (*I*² = 83.0%) (Fig. 6).

### Quality of life metrics

**Figure 7:**
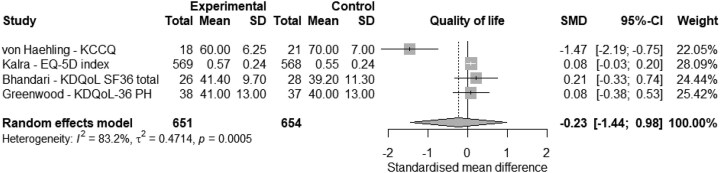
Standardized mean difference of pooled quality of life metrics.

Four studies reporting validated QoL outcomes were included in the random-effects SMD meta-analysis (Table 3). One overall or most clinically relevant QoL measure was selected from each study. There was no significant difference in QoL between IV iron and control groups (SMD −0.23, 95% CI −1.44 to 0.98; *P* = .587), with substantial heterogeneity observed (*I*² = 83.2%) (Fig. 7).

**Table 3:** Summary of quality-of-life outcomes reported across four studies evaluating intravenous iron in non-dialysis CKD with iron deficiency.

Study	Measure	IV iron (Mean, Range/SD, MD)	Control (mean, range/SD, MD)	*P* value
von Haehling *et al*. (2024)	EQ-5D-3 L index	0.68 (0.50–0.78)	0.69 (0.60–0.78)	.63
		+0.06	+0.01	
	KCCQ	60 (56–81)	70 (54–82)	.21
		+10	−2	
Kalra *et al*. (2022)	EQ-5D index	0.57 (0.01)	0.55 (0.01)	.57
		−0.04	−0.05	
	EQ-5D VAS	59.9 (1.3)	59.4 (1.3)	.75
		−3.3	−3.6	
Bhandari *et al*. (2021)	KDQOL-SF36 total	41.4 (9.7)	39.2 (11.3)	.892
		+1.2	−1.1	
	KDQOL-SF36 physical health	39.1 (9.4)	36.8 (11.9)	.823
		−0.1	−1.7	
	KDQOL-SF36 mental health	44.6 (10.6)	42.8 (10.3)	.936
		+2.3	+0.6	
Greenwood *et al*. (2023)	KDQOL-36 physical health	41 (13)	40 (13)	.82
		+1	+0	
	KDQOL-36 mental health	45 (10)	48 (11)	.055
		+1	+6	
	KDQOL-36 burden	73 (23)	64 (32)	.866
		+5	+2	
	KDQOL-36 symptoms	77 (16)	76 (22)	.871
		+7	+2	
	KDQOL-36 effects	81 (19)	79 (25)	.601
		+3	+1	

Across all instruments (EQ-5D-3 L, EQ-5D VAS, KCCQ, KDQOL-SF36/36). EQ-5D, EuroQol 5-Dimension; VAS, visual analogue scale; KCCQ, Kansas City cardiomyopathy questionnaire; KDQOL; kidney disease quality of life; MD, mean difference; QoL, quality of life.

**Figure 8:**
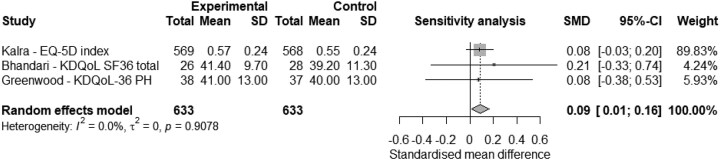
Sensitivity analyses of mean difference of pooled quality of life metrics.

Sensitivity analysis excluding von Haehling *et al*., in which SDs were estimated from reported ranges, demonstrated a small statistically significant effect favouring IV iron (SMD 0.09, 95% CI 0.01–0.16; *P* = .037), with no observed heterogeneity (*I*² = 0%). Given the small effect size and variation in QoL instruments, this finding should be interpreted cautiously (Fig. 8).

### Adverse events

There is generally no significant difference to the safety profiles of those taking both the IV iron preparations and those receiving the placebo/usual care across multiple studies (Table 4). Most common adverse events were those of a CV or infective nature but incidence of these were comparable across groups. McMahon *et al*. did not record adverse events in their study.

**Table 4:** Detail of reported adverse events.

Study	Intervention	Control	Adverse event
Von Haehling *et al*. (2024)	16 (81%)	17 (88%)	Number of patients with at least one adverse event showed no significant difference between placebo and intervention (*P* = 1.00). Results for serious adverse events (19 vs 5) were primarily driven by adverse events that required inpatient hospitalization/prolonging of existing hospitalization (16 vs 4 events) and by events that were deemed life-threatening (3 vs none). There were no fatalities in either group.
Kalra *et al*. (2022)	410 (73%)	435 (77%)	There was no significant difference (*P* = .21) in terms of adverse events between groups. Most commonly serious adverse events were cardiac or infective. There was 103 (20%) versus 115 (21%) all-cause mortality rate (intervention vs control, *P* = .48).
Bhandari *et al*. (2021)	9 (47.4%)	10 (52.6%)	There were no treatment-related serious adverse events—no deaths, strokes, or hospitalizations. There were also no hypersensitivity reactions or infusion reactions with FDI. The most common adverse event was an infection.
Greenwood *et al*. (2023)	3 (7.9%)	5 (13.5%)	No adverse events attributable to the iron therapy. Most common adverse event was infections.
Al Sahow *et al*. (2022)			57/289 (19.7%) hospital admissions over 12 months, 88% for unspecified non-infective reasons.

## DISCUSSION

Findings from this systematic review indicate that IV iron is biologically active but clinically unproven in people with ND-CKD who are iron deficient but not anaemic. Our study had shown that IV iron improves biochemical markers of ID, however, has no demonstratable or measurable patient benefit in terms of symptomatic relief, improved QoL, or physical functioning. Secondary outcomes including the 6MWT, QoL, and adverse outcomes, demonstrated no significant differences in all reported metrics between both intervention and control groups.

IV iron significantly improved Hb, TSAT, and ferritin in four studies (McMahon, Bhandari, Greenwood, and von Haehling). There were consistent improvements shown across all trials and these findings are biologically plausible and expected given the intervention, though there is limited novelty given the already established iron physiology and prior ND-CKD literature. ID is a well-documented phenomenon in ND-CKD and studies have indicated the benefit (and risk) of IV iron, but guidelines are inconsistent in their recommendations and generally do not address those without anaemia, with KDIGO suggesting treatment in this population in extreme deficiency [24].

Importantly, improvements in circulating iron indices did not translate into measurable functional benefit. This dissociation may reflect CKD-specific pathophysiological mechanisms beyond absolute ID. Chronic low-grade inflammation is common in ND-CKD and drives increased hepatic production of hepcidin, the principal regulator of iron homeostasis. Elevated hepcidin suppresses ferroportin-mediated iron export from enterocytes and macrophages, promoting reticuloendothelial iron sequestration and limiting iron bioavailability to peripheral tissues despite apparently adequate ferritin and TSAT levels [44]. Consequently, biochemical correction of iron parameters may not necessarily reflect restoration of effective intracellular iron utilization within skeletal muscle or other metabolically active tissues. In addition, CKD-associated inflammation, uraemic toxin accumulation, endothelial dysfunction, sarcopenia, and mitochondrial impairment may independently contribute to fatigue and reduced exercise capacity, potentially limiting the symptomatic impact of iron repletion alone [45]. This may help explain why improvements in ferritin and TSAT were consistently observed without corresponding gains in QoL or physical performance outcomes.

A key finding from this study is a clear lack of demonstrable benefit in physical function. In the four trials measuring the 6MWT, no significant difference was shown: pooled random-effects MD was 3.40 m (95% CI −60.80 to 67.60; *P* = .917), with a high heterogeneity (*I*² = 0.9%; *P* < .001) and wide confidence intervals, with highly variable and inconsistent results across the trials. We propose multiple competing explanations for this conclusion: there is indeed a true lack of functional benefit of IV iron in non-anaemic ND-CKD; there are limitations to outcome measurements themselves (functionally there is a high baseline capacity and ceiling in the ND-CKD populations) or there is insufficient power and a short follow-up in respect to all of the pooled studies (total participants across all studies: 1695, total with final analysis outcome: 942). Ultimately, there is an unresolved uncertainty rather than a definitive negative conclusion, when considering the benefits of IV iron in terms of improvement in physical function.

It was not possible to estimate the impact on symptomatic burden and QoL due to the marked variability in measurement tools used across studies. The lack of standardized approaches prevented meaningful pooling of data and contributed to reliance on small sample sizes and short intervention periods. Four studies reported validated QoL indicators; however, none used the same tool or a directly comparable measure within the same instrument. This heterogeneity may also suggest that standardized QoL tools lack sensitivity to detect modest functional or symptomatic improvements in iron-deficient but not anaemic CKD populations. Furthermore, fatigue and exercise intolerance in CKD are multifactorial and may not be substantially altered by correction of biochemical ID alone.

There are also concerns about the safety of IV iron in ND-CKD without anaemia. Our analyses have suggested no clear signal of harm across all of our included studies. CV and infective events were comparable between both intervention and control groups, with these being not significantly different in studies which had carried out analyses. All trials which reported adverse events reported none or comparable: deaths, serious adverse events, or severe allergic reactions, suggesting serious reactions are rare. This reassurance, however, may be tempered by limited event numbers and a short follow-up period and there is a clear absence of robust data on long-term safety and cumulative dosing. Other studies reinforce the indicated safety profile of IV iron preparations in CKD, though none with a directly comparable population in which non-anaemic and non-dialysis status is specified [23, 34, 36, 46–49].

Several limitations should be considered. Most included studies were small, with five of six enroling fewer than 100 participants, limiting power for patient-reported and functional outcomes. There was also substantial heterogeneity in ND-CKD stage, definitions of ID, IV iron dosing regimens, follow-up duration, and outcome reporting. Trials frequently prioritized biochemical endpoints, while functional outcomes, QoL, symptom burden, CV events, and infection outcomes were reported inconsistently. This limited the ability to pool non-biochemical outcomes and leaves residual uncertainty despite consistent improvements in haematological markers.

Variation in iron-deficiency definitions was an important additional source of heterogeneity. Studies used differing thresholds based on ferritin, TSAT, reticulocyte Hb content, and percentage hypochromic red cells. These markers reflect related but distinct aspects of iron status: ferritin may indicate iron stores but is confounded by inflammation in CKD; TSAT reflects circulating iron availability but is biologically variable; and cellular indices may better capture functional iron restriction. These differences may have influenced treatment response, with some studies enroling participants with absolute ID and others including those with inflammation-mediated iron restriction.

Formal subgroup analysis by iron-deficiency definition was not performed because of the small number of studies, overlapping eligibility criteria, and concurrent variation in dosing, follow-up, and outcome selection. Such an analysis would have been underpowered and at risk of ecological bias. Heterogeneity in iron-deficiency definitions should therefore be considered an important limitation and a potential contributor to the uncertainty around patient-centred outcomes.

### Implications for clinical practice

Current evidence from this review supports using IV iron in non-anaemic ND-CKD for biochemical correction but does not indicate routine clinical use for symptomatic or functional improvement. However, this review does reinforce the conservative guideline positions with NICE having no specific guidelines [15] and KDIGO only recommending IV iron in profound deficiency [24].

Further research is needed to directly target outcomes such as physical function, fatigue, symptom burden, or CV events, these should be of adequate power and include long-term follow up. From this we would recommend adoption of uniform, kidney-relevant outcomes, aligned with the SONG initiatives [14] and avoidance of heterogenous QoL instruments across future trials. Moreover, in future trials, there should be a clear target population with defined phenotyping of ID without anaemia, this may help identify the subgroups most likely to benefit from IV iron.

## CONCLUSION

In conclusion, the available evidence suggests that IV iron improves biochemical iron indices in ND-CKD without anaemia, however, there is no convincing evidence of benefit in functional outcomes, QoL, or clinical endpoints. Safety cannot be considered established: adverse event data are limited, follow-up is short and event rates are low. As such, the long-term safety profile remains uncertain, particularly with respect to CV and infection outcomes. High-quality, adequately powered trials with sufficient follow-up and a focus on patient-important outcomes are urgently required.

## Data Availability

The data underlying this article will be shared on reasonable request to the corresponding author.
